# Supplementary material for: Mapping urban gullies in the Democratic Republic of the Congo
Source: Nature. 2025 Aug 27;644(8078):952–9. doi: 10.1038/s41586-025-09371-7 (PMC12390838; doi:10.1038/s41586-025-09371-7)
Supplement: Supplementary file 1 — This file contains Supplementary Figs. and Supplementary Tables that provide further details, examples and data on the mapping of UGs across the DRC; the construction and application of the UG susceptibility model; the assessment of the population displaced by UG formation and expansion; the estimated exposed population; the uncertainties in our estimates of displaced and exposed population; and the interpretation of the results. [file 41586_2025_9371_MOESM1_ESM.docx]

**SUPPLEMENTARY INFORMATION**

**
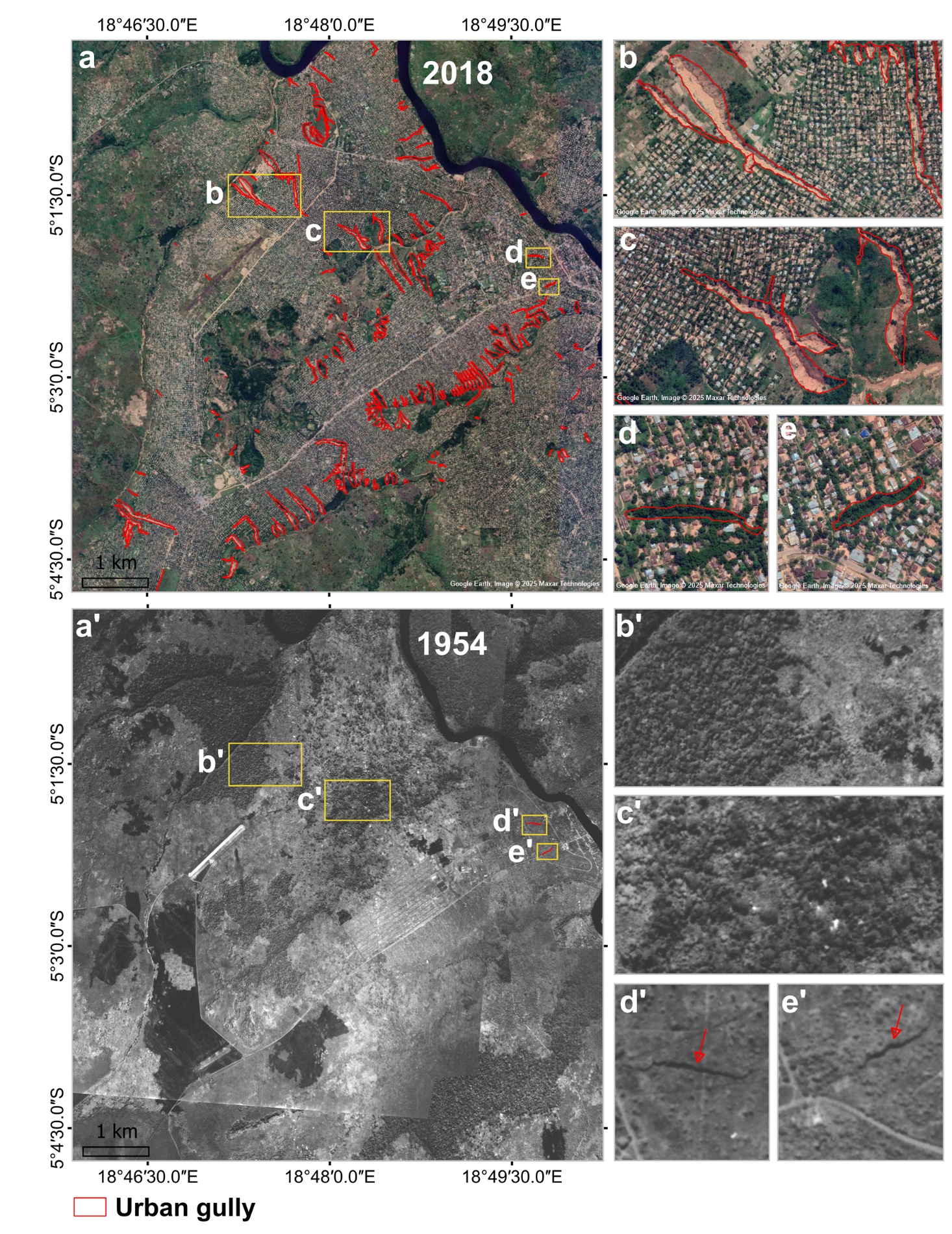
**

**Supplementary Fig. 1-1 |** Example of the verification of the presence of urban gullies (UGs) in the 1950s for the city of Kikwit. **(a), (b), (c), (d)** and **(e)** show the mapped extent of UGs in 2018. The background is a satellite image of 24/05/2018 (Google Earth, Image © 2025 Maxar Technologies). Subfigures **(a’), (b’), (c’), (d’)** and **(e’)** show the same locations on panchromatic aerial photographs of 1954. One can observe that the city extent was much more limited. Only two of the mapped UGs were already visible (cf. **d & e**). Both gullies are linked to the road network and this early urbanization.


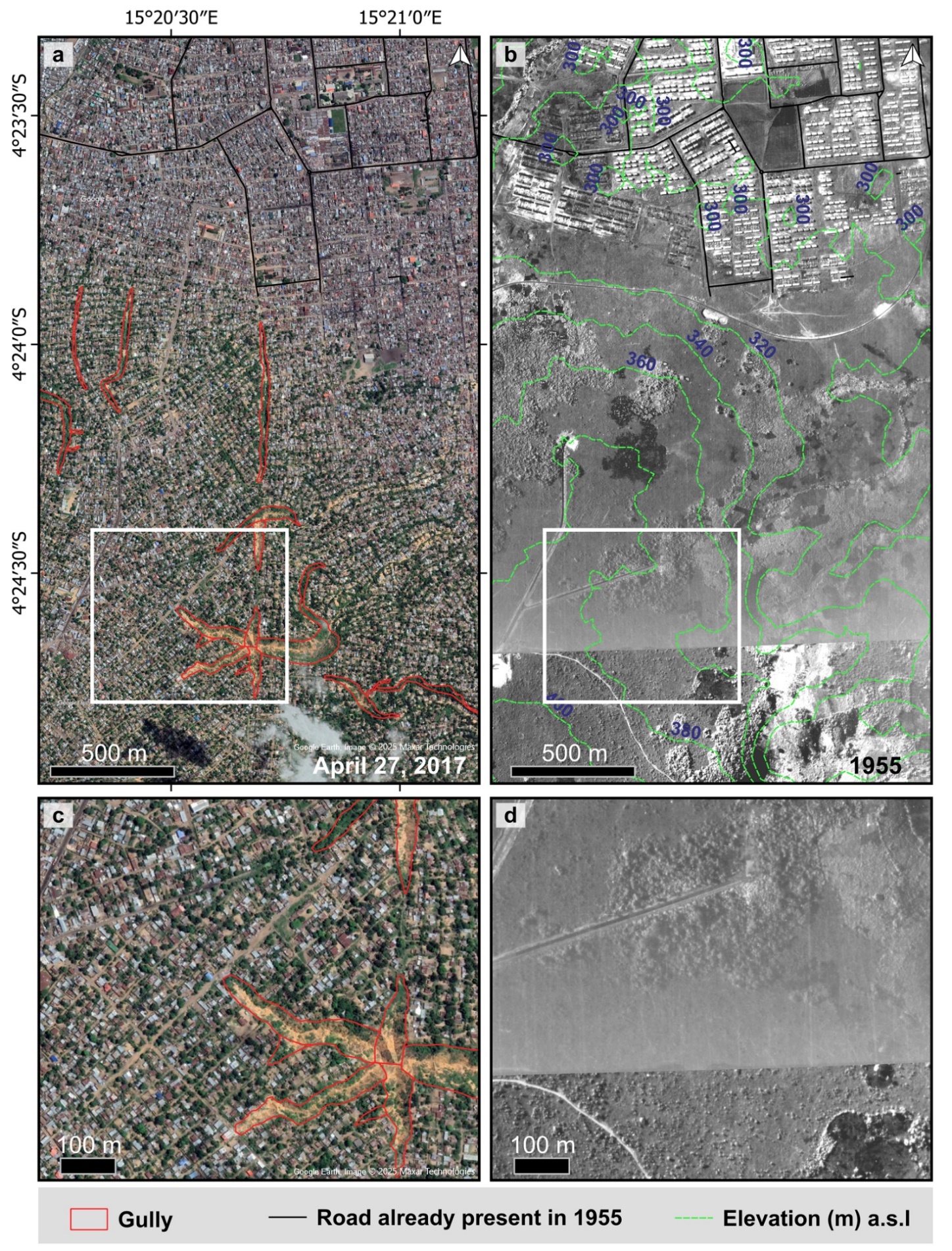


**Supplementary Fig. 1-2 |** Example of the verification of the presence of urban gullies (UGs) in the 1950’s for the city of Kinshasa. **(a)** and **(c)** show a satellite image of 27/04/2017 (Google Earth, Image © 2025 Maxar Technologies) of parts of Kinshasa with the extent of urban gullies mapped in red. **(b)** and **(d)** show1955 panchromatic aerial photograph of the same areas. In 1955, the flat area situated to the north (commune of Matete) is already urbanized, while the steeper area to the south (commune of Kinsenso) is not. No gullies are observed in 1955.

**
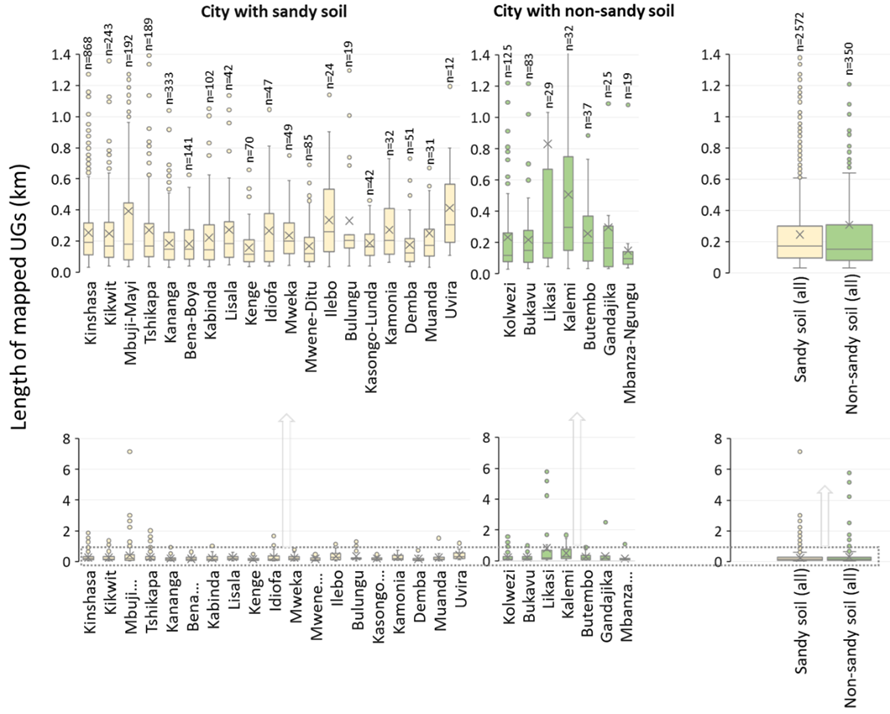
**

**Supplementary Fig. 2-1 |** Boxplots of the length of all observed urban gullies (UGs) as mapped on the reference date image. Data are grouped per city and based on whether the city is located on sandy or non-sandy soil substrates. The lower plots show the full range of all observations, while the upper plots provide a zoom of the most common range.


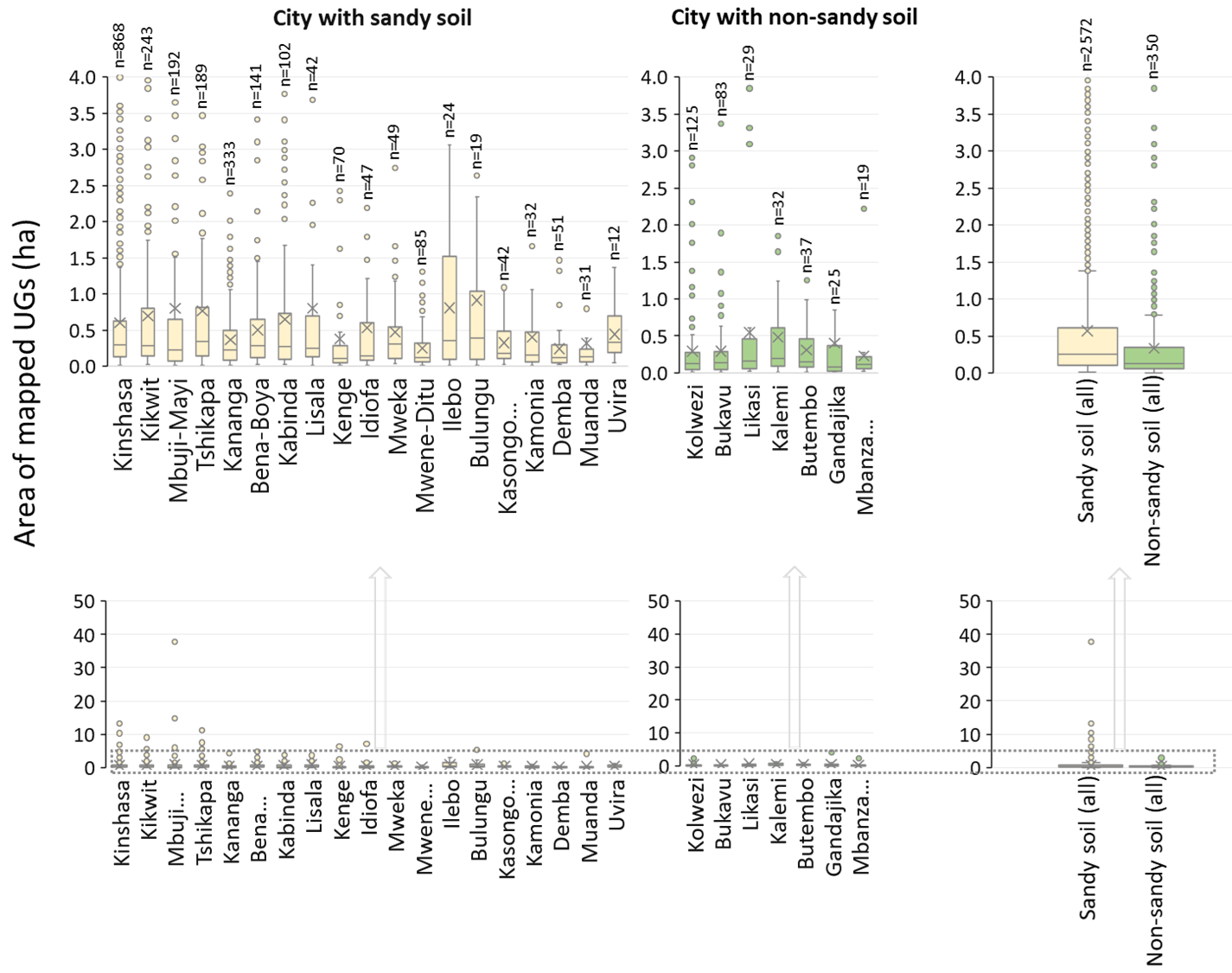


**Supplementary Fig. 2-2 |** Boxplots of the areal extent of all observed urban gullies (UGs) as mapped on the reference date image. Data are grouped per city and based on whether the city is located on sandy or non-sandy soil substrates. The lower plots show the full range of all observations, while the upper plots provide a zoom of the most common range.

**
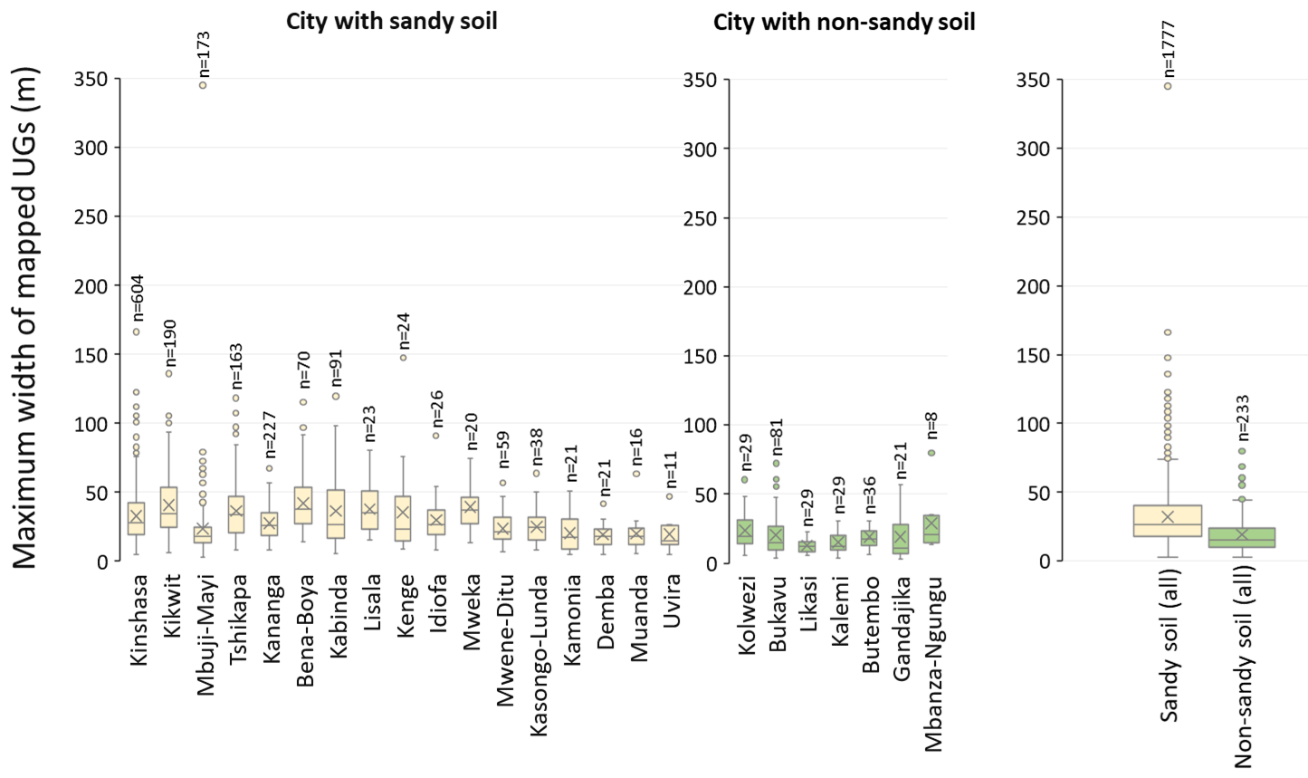
**

**Supplementary Fig. 2-3 |** Boxplots of the maximum width of urban gullies (UGs) as mapped on the reference date image. Boxplots include all UGs with an age of at least ten years. Data are grouped per city and based on whether the city is located on sandy or non-sandy soil substrates.

**
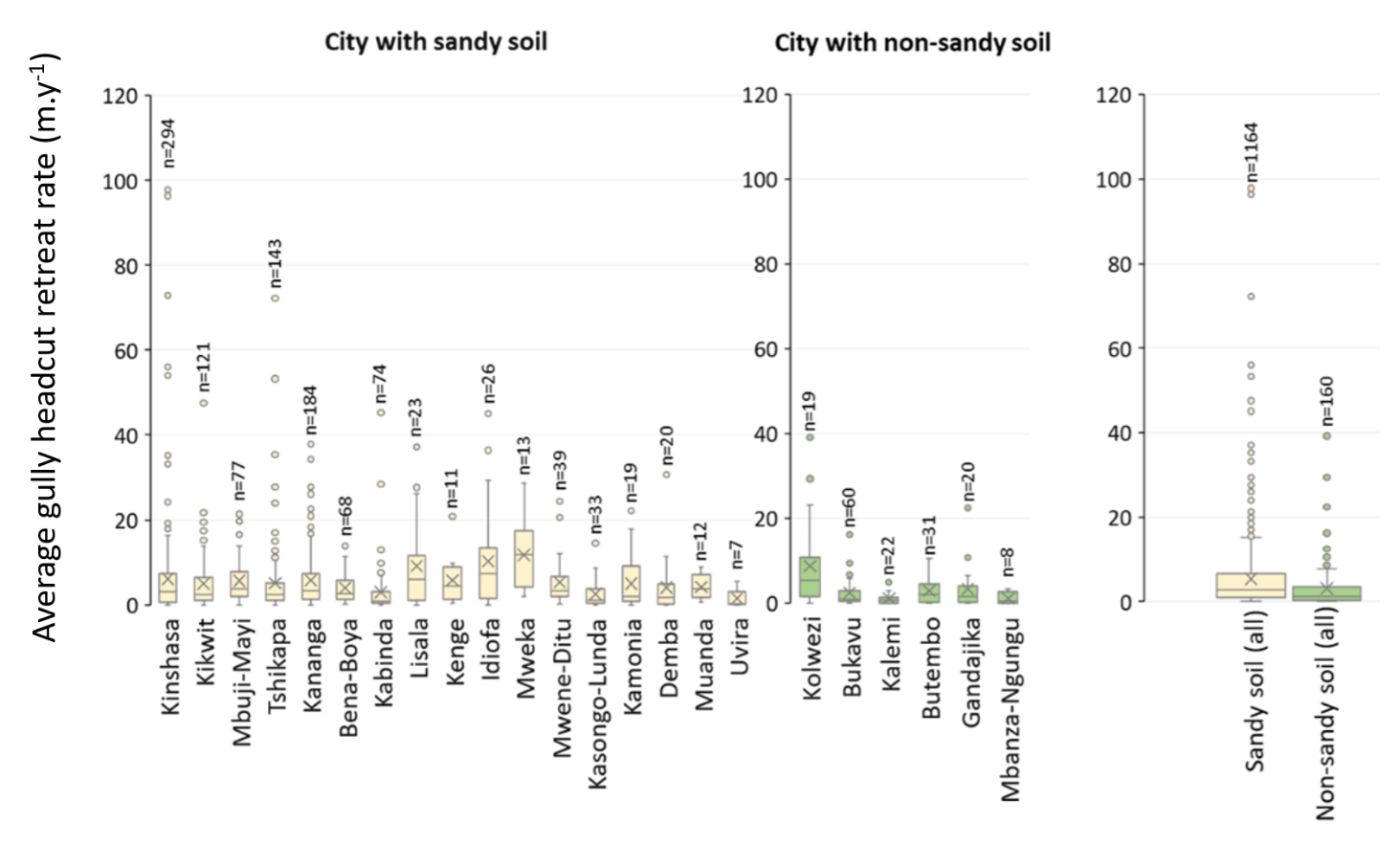
Supplementary Fig. 2-4 |** Boxplots of the long-term average linear gully head retreat rate of (UGs) for all UGs with an observation period of at least ten years. The linear retreat is quantified as the Euclidean distance between gully heads as measured on the oldest and most recent suitable image available. Data are grouped per city and based on whether the city is located on sandy or non-sandy soil substrates.


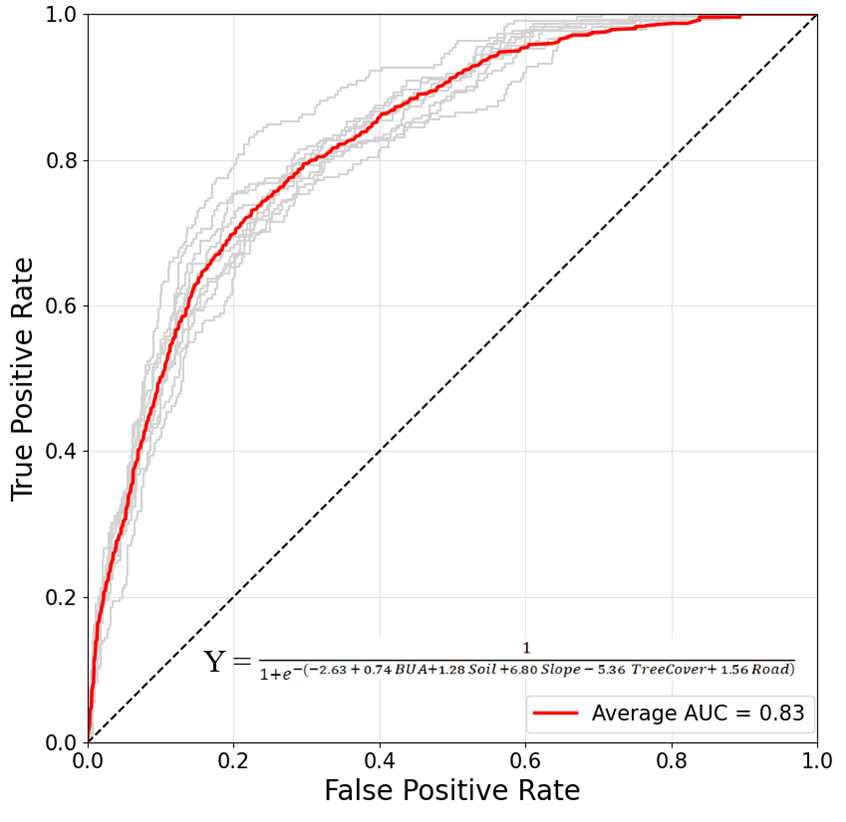


**Supplementary Fig. 3 |** Receiver Operating Characteristic Curve (ROC) of a logistic regression model simulating the susceptibility to urban gullying in and around Congolese cities at a resolution of 30 arcseconds. The red curve indicates the average ROC and corresponding Area Under the Curve (AUC) for ten Monte Carlo cross validations (indicated in grey; cf. **Methods, ‘Assessing the factors controlling UG occurrence’**). The equation shows the final model, trained on all data, where Y is the simulated susceptibility to (urban) gullies, ‘BUA’ is the standardized fraction of built-up area; ‘Soil’ is a dummy variable indicating whether the pixel is located in dominantly sandy soils (1) or not (0); ‘Slope’ is the standardized average slope steepness within the pixel; ‘TreeCover’ is the standardized fraction of tree cover; and ‘Road’ is the standardized road density within the pixel. Further information on these variables is provided in **Supplementary Table 2**. **Supplementary Table 3** provides further details on the fitted coefficients.


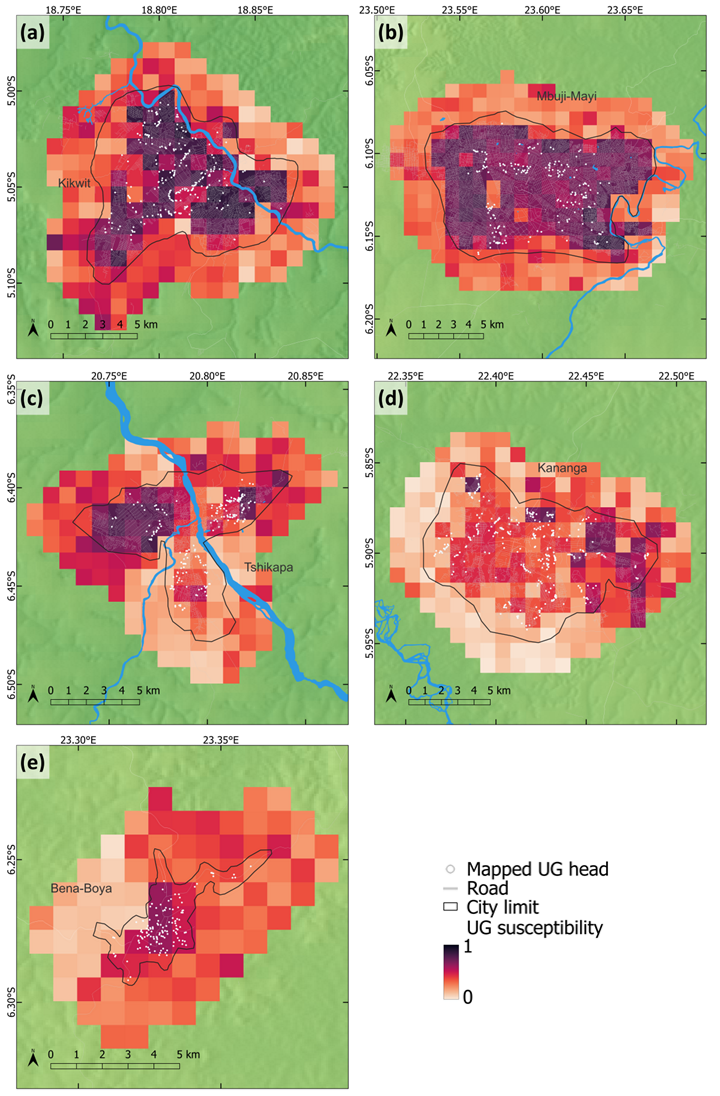


**Supplementary Fig. 4 |** Maps of the urban (UG) gully susceptibility model (cf. **Supplementary Fig. 3**) applied to the Congolese cities where UGs were observed (cf. **Supplementary Table 1**) as well as their surrounding areas. The spatial resolution of the model is 30 arcseconds. Results for Kinshasa are shown in **Fig. 3b**. **(a)** Kikwit. **(b)** Mbuji-Mayi. **(c)** Tshikapa. (**d**) Kananga. (**e**) Bene-Boya. (**f**) Kabinda. (**g**) Kolwezi. (**h**) Lisala. (**i**) Kenge. (**j**) Idiofa. (**k**) Bukavu. (**l**) Mweka. (**m**) Mwene-Ditu. (**n**) Ilebo. (**o**) Bulungu. (**p**) Likasi. (**q**) Kalemi. (**r**) Kasongo-Lunda. (**s**) Kamonia. (**t**) Demba. (**u**) Butembo. (**v**) Gandajika. (**w**) Muanda. (**x**) Uvira. **(y)** Mbanza-Ngungu.

**
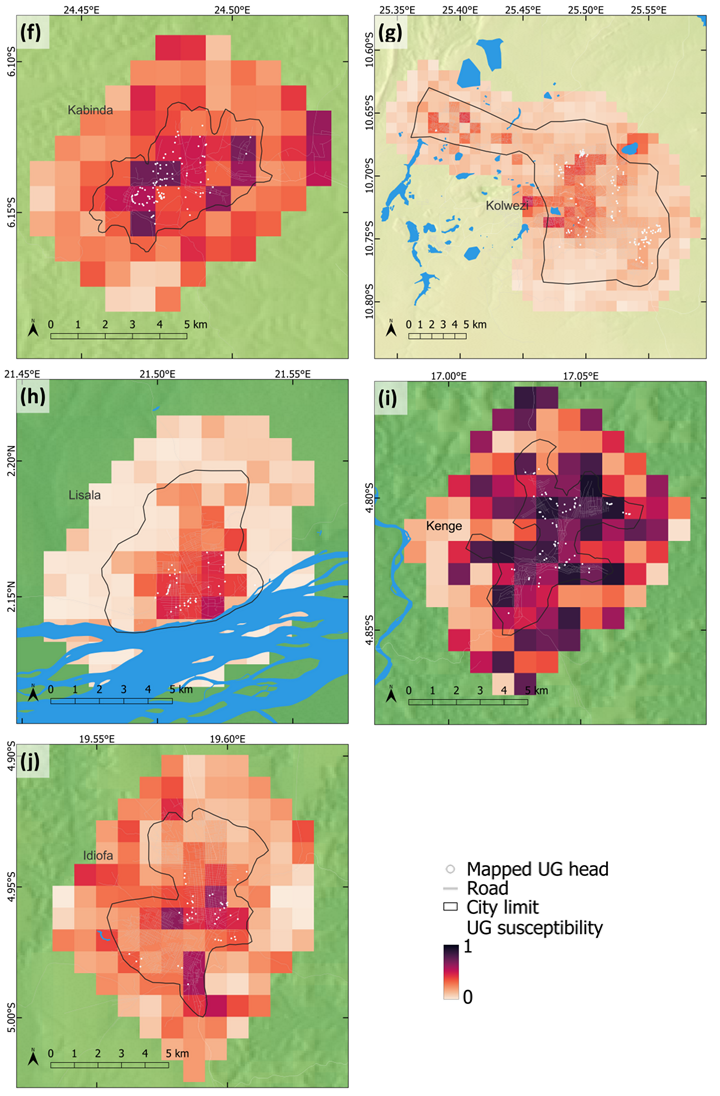
**

**Supplementary Fig. 4 |** Continued.

**
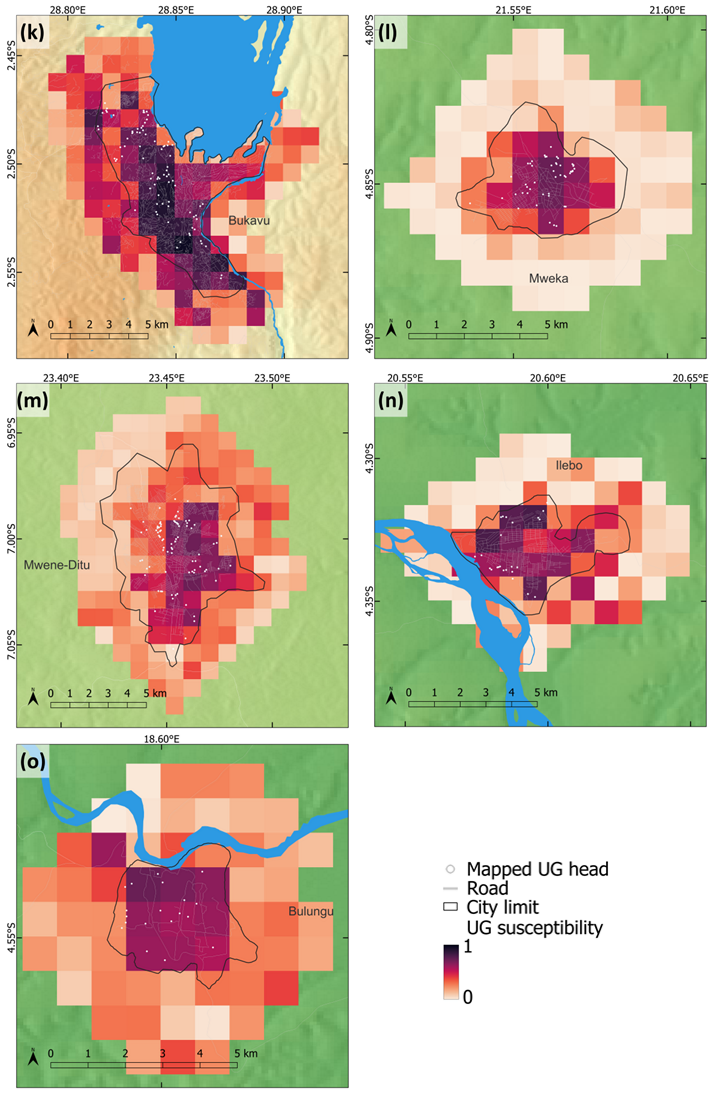
**

**Supplementary Fig. 4 |** Continued.

**
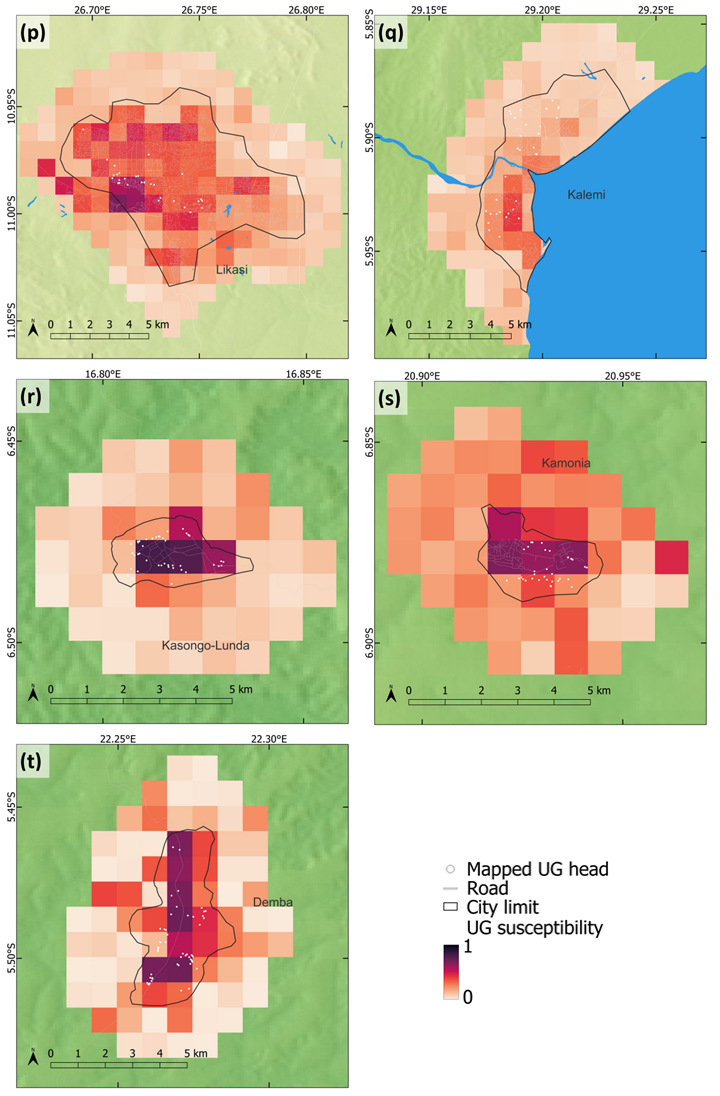
**

**Supplementary Fig. 4 |** Continued.


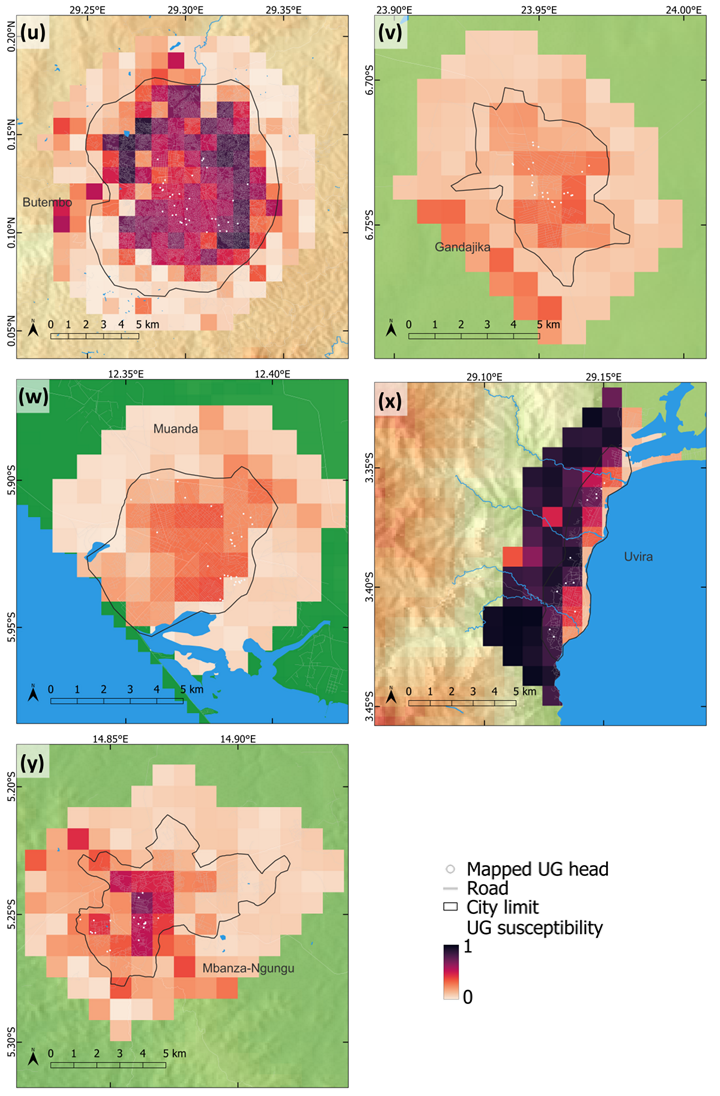


**Supplementary Fig. 4 |** Continued.


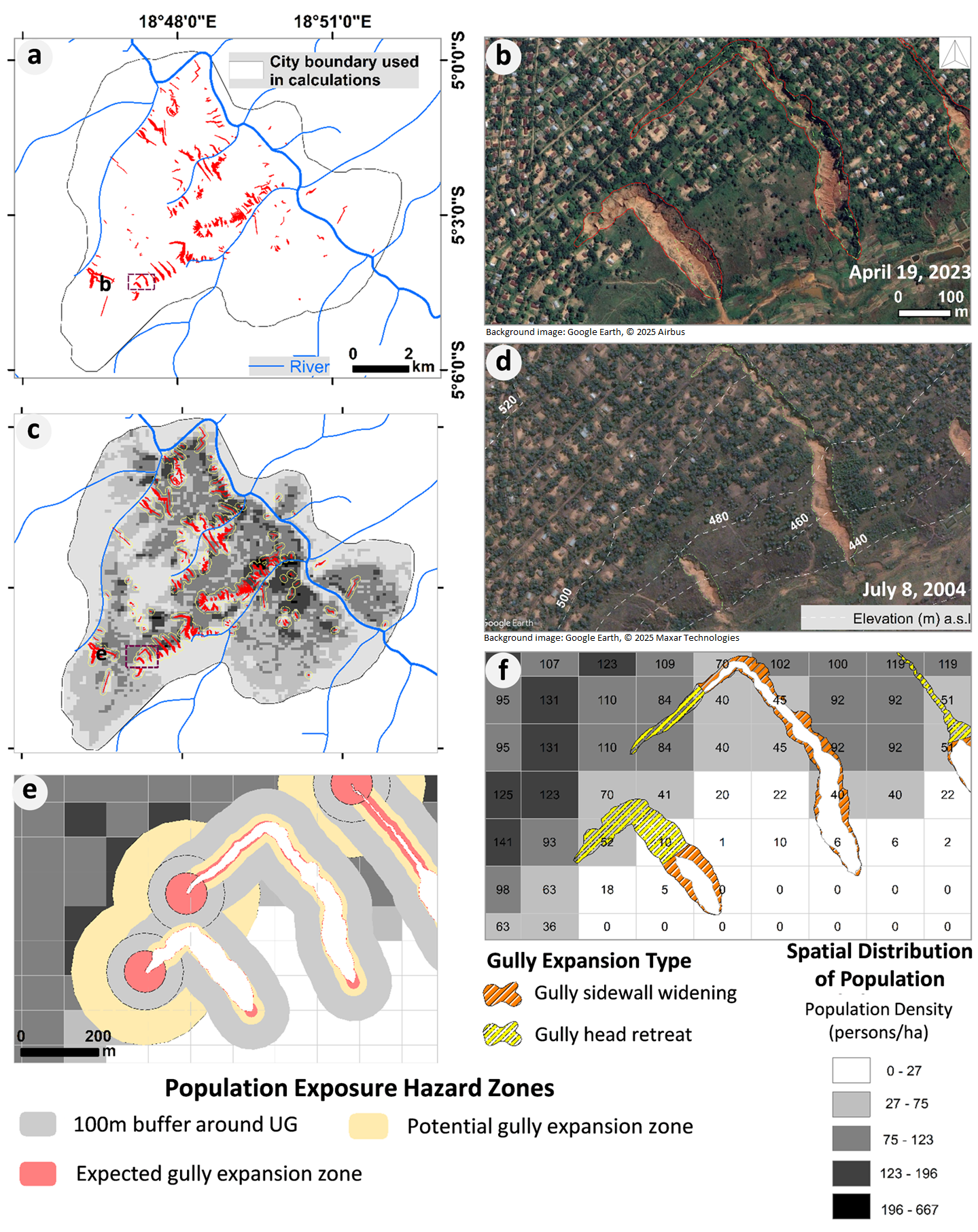


**Supplementary Fig. 5 |** Illustration of the geoprocessing operations conducted to quantify the population displaced by or exposed to urban gully (UG) expansion, using the city of Kikwit as example. **(a)** Inventory of all mapped UGs, using a recent Google Earth image as reference image (taken on 19/04/2023; cf. **Supplementary Table 1**). The indicated city boundary was delineated visually on the same image and correspond to the zone that was carefully checked for the presence of UGs. **(b)** Close-up of the extent of some UGs as mapped on the reference image (in red). **(d)** The corresponding UG extents as they appear on the first available image (green dashed line). Note that these UGs initially expand along the line of steepest slope but, especially at later stages, their further expansion is also influenced by the layout of the road network. **(c)** JRC GHS Population raster data, indicating the estimated population density at a resolution of 100 m for the year 2023^8^, overlayed with the mapped UGs. **(e)** Close-up illustrating the delineation of different hazard zones of exposed population (cf. **Methods, ‘Estimating the exposed population’**). **(f)** Close-up illustrating the calculation of gully expansion rates and corresponding displaced population due to sidewall widening and gully head retreat (cf. **Methods, ‘Estimating the displaced population’**).

**
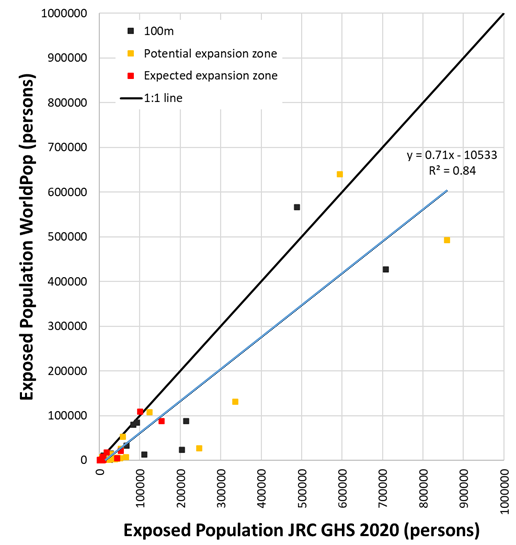
**

**Supplementary Fig. 6 |** Comparison of the exposed population in different hazard zones in 2020 as estimated based on WorldPop^66,67^ and JRC GHS^8^ population density data. Each point corresponds to the total population living in the indicated hazard zone of one of the 26 cities affected by urban gullies (cf. **Fig. 1; Supplementary Table 6**). The regression line indicates the average correlation for all cities and hazard zones (n=78).

**
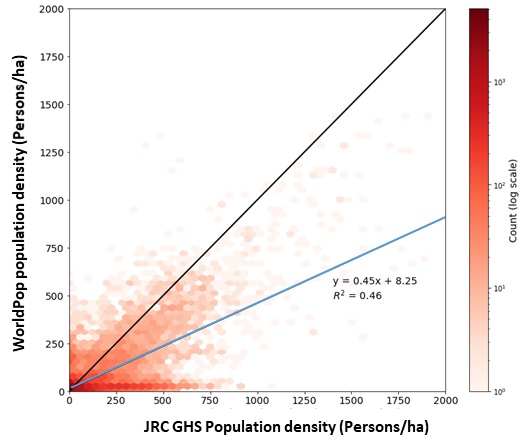
**

**Supplementary Fig. 7 |** Relationship between WorldPop^66,67^ and JRC GHS^8^ population density data for 21,202 points across all cities of the D.R. Congo that are significantly affected by urban gullies (cf. **Fig. 1**). Data points were sampled randomly and proportional to the size of each city for the year 2020. Note the large amount of data points where JRC GHS assumes a considerable population density but WorldPop provides an estimate close to zero. Given that points were sampled within (typically densely populated) cities, WorldPop values are likely underestimated.


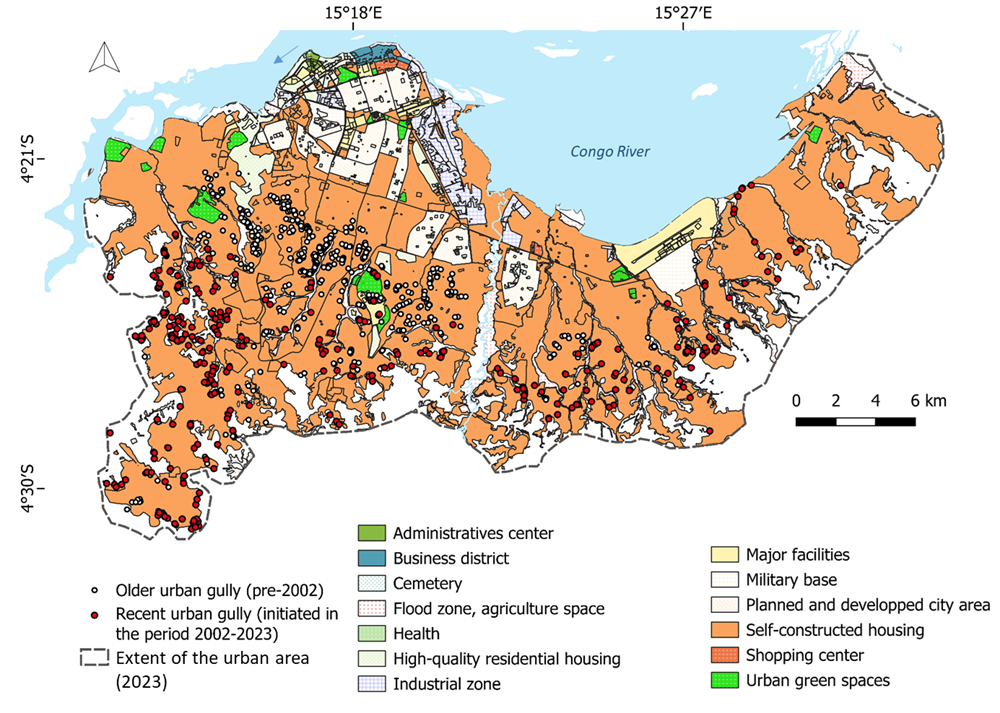


**Supplementary Fig. 8 |** Comparison of mapped urban gully head locations in Kinshasa and the different land use zones of the Kinshasa Metropolitan Area Strategic Master Plan (based on^11^). Nearly all urban gullies are located in ‘self-constructed housing’ zones that grew spontaneously and without further urban planning. Also note that older urban gullies already present on the first available Google Earth images (2002, cf. **Supplementary Table 1**) are typically closer to the planned and developed city center, providing a further indication of the link between unplanned urban sprawl and urban gully formation.


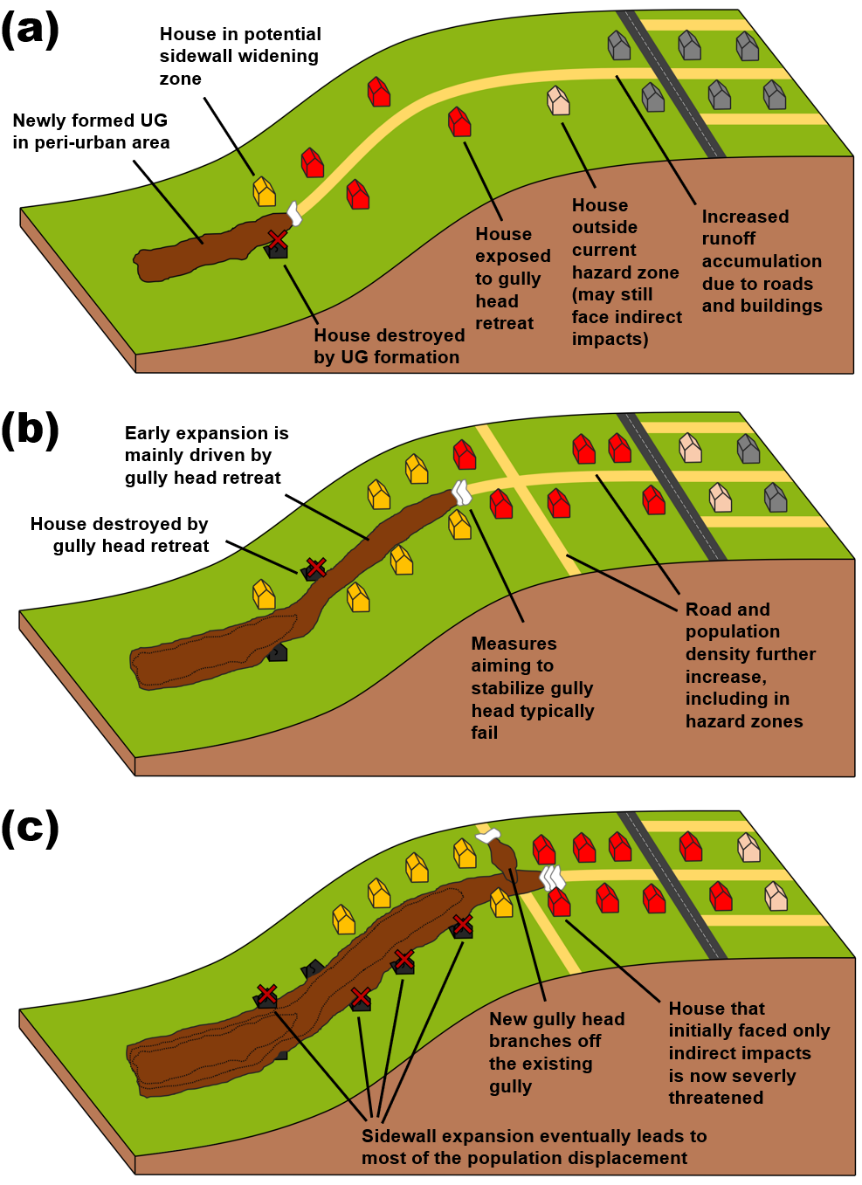


**Supplementary Fig. 9 |** Conceptual model illustrating the effect of urban gully expansion on population exposure and displacement. **(a)**, **(b)** and **(c)** show respectively an early, middle and late stage of urban gully development.

**Supplementary Table 1 |** Overview of the inventory urban gullies (UGs) constructed for the 26 significantly affected cities (ordered based on the total areal extent of UGs on the Reference Image). GE = Google Earth; RI = Reference Image; # = number. Note that maximum gully width values were only determined for UGs with an observation period of at least ten years.

**Supplementary Table 2 |** Overview of the potential predictor variables considered for the construction of the urban gully susceptibility model (cf. **Fig. 3, Supplementary Fig. 3, Supplementary Fig. 4, Supplementary Table 3**).

| Variables | Description | Units | Observed Range |  | Original Resolution | Source |
| --- | --- | --- | --- | --- | --- | --- |
| Slope | Average slope steepness based on 90 m SRTM4.1 data | ° | 0 – 20.06 |  | 30 arcseconds | ^55^ |
| Rainday13 | Average number of days with rainfall above 13 mm/day (1979–2021) | days | 12.33 – 40.88 |  | 0.1° | ^56,57^ |
| Rainday20 | Average number of days with rainfall above 20 mm/day (1979–2021) | days | 2.52 –15.88 |  | 0.1° | ^56,57^ |
| Rain_mean | Mean annual rainfall (1979–2021) | mm | 818.53 – 1,922.71 |  | 0.1° | ^56,57^ |
| RDN | Rainy Day Normal, i.e. the average amount of rain on a day with rainfall (1979–2021) | mm/day | 2.87 – 6.31 |  | 0.1° | ^56,57^ |
| Max_Daily | Maximum Daily Rainfall (1979–2021) | mm/day | 39.13 – 266.38 |  | 0.1° | ^56,57^ |
| K | Soil erodibility, i.e. the K-factor as derived in the Revised Universal Soil Loss Equation | Mg.ha.h/ (ha.MJ.mm) | 0.007 –0.034 |  | 250 m | ^58^ |
| Soil | Dummy variable indicating whether the pixel is located on the Kwango-Kwilu and Kasai Plateaus and likely has soils formed on sandy parent material (1) or not (0) |  | 0 – 1 |  |  | ^49^ |
| Sand_iSDA | Sand content in the topsoil (0–20 cm) | % | 31.38 – 76.72 |  | 30 m | ^59^ |
| Tree_Cover | Percentage of tree cover | % | 0 – 100 |  | 100 m | ^60^ |
| BUA | Percentage of built-up area in 2019 | % | 0 – 100 |  | 100 m | ^60^ |
| Road | Road density according to Open Street Map | m/km² | 0 – 32,367 |  | 100 m | ^61^ |

**Supplementary Table 3 |** Details on the fitted coefficients of the logistic regression equation used to simulate urban gully susceptibility in and around Congolese cities (cf. **Fig. 3, Supplementary Fig. 3, Supplementary Fig. 4**). The ‘95% Confidence Interval’ indicates the confidence interval on the coefficients for the final model (trained on all data). ‘Coefficient Range MC simulation’ indicates the range in fitted coefficients over ten Monte Carlo simulations where the dataset was each time split in 70% for training and 30% for testing. All variables were each time standardized for the range of the training data (cf. **Methods, ‘Assessing the factors controlling UG occurrence’**).

| Variable | Coefficient | standard error | z | p-value | 95% Confidence Interval | Coefficient Range MC simulation |
| --- | --- | --- | --- | --- | --- | --- |
| Slope | 6.8027 | 0.527 | 12.897 | <0.0001 | 5.769 – 7.837 | 5.663 – 7.469 |
| Soil | 1.2773 | 0.112 | 11.435 | <0.0001 | 1.058 – 1.496 | 1.06 – 1.477 |
| Tree_cover | -5.3587 | 0.776 | -6.906 | <0.0001 | -6.88 – -3.838 | -6.163 – -5.241 |
| BUA | 0.7367 | 0.181 | 4.072 | <0.0001 | 0.382 – 1.091 | 0.542 – 0.899 |
| Road | 1.556 | 0.309 | 5.032 | <0.0001 | 0.95 – 2.162 | 1.246 – 1.958 |
| constant | -2.6337 | 0.168 | -15.639 | <0.0001 | -2.964 – -2.304 | -2.881 – 2.459 |

**Supplementary Table 4 |** Overview of the mapped expansion of urban gullies and the associated estimated displaced population per affected city. A distinction is made between expansion and displacement due to the formation of new gullies, gully head retreat and gully sidewall widening (cf. **Methods, ‘Estimating the displaced population’; Supplementary Fig. 5f**). ‘Total Population in year of RI’ indicates the total population living within the mapped city extent according to the JRC GHS dataset^8^ in the year of the Reference Image (RI; cf. **Supplementary Table 1**).

**Supplementary Table 5-1 |** Overview of the population per city, living less than 100 m away from an urban gully in the indicated year (cf. **Methods, ‘Estimating the exposed population’**). Cities are ordered based on the total spatial extent of urban gullies on the date of the reference image (cf. **Supplementary Table 1**).

**Supplementary Table 5-2 |** Overview of the population per city, living in the potential expansion zone of an urban gully in the indicated year (cf. **Methods, ‘Estimating the exposed population’**). Cities are ordered based on the total spatial extent of urban gullies on the date of the reference image (cf. **Supplementary Table 1**).

**Supplementary Table 5-3 |** Overview of the population per city, living in the expected expansion zone of an urban gully in the indicated year (cf. **Methods, ‘Estimating the exposed population’**). Cities are ordered based on the total spatial extent of urban gullies on the date of the reference image (cf. **Supplementary Table 1**).

**Supplementary Table 6 |** Comparison of the exposed population living in different hazard zones in different cities, based on JRC GHS population density data^8^ and WorldPop population density data^66,67^ for 2020 (cf. **Methods, ‘Uncertainty assessment’; Supplementary Fig. 6**). Relative differences are calculated as the WorldPop estimate minus the JRC GHS estimate, divided by the JRC GHS estimate.

| N° | City | JRC GHS 2020 | | | WorldPop 2020 | | | Relative difference | | |
| --- | --- | --- | --- | --- | --- | --- | --- | --- | --- | --- |
|  |  | 100m | Potential expansion zone | Expected expansion zone | 100m | Potential expansion zone | Expected expansion zone | 100m | Potential expansion zone | Expected expansion zone |
| 1 | Kinshasa | 711377 | 875286 | 140051 | 425434 | 495549 | 81194 | 40.2% | -43.4% | -42.0% |
| 2 | Kikwit | 85498 | 126334 | 16686 | 80454 | 108038 | 15921 | 5.9% | -14.5% | -4.6% |
| 3 | Mbuji-Mayi | 487098 | 584615 | 96718 | 564440 | 637732 | 109235 | -15.9% | 9.1% | 12.9% |
| 4 | Tshikapa | 202269 | 242729 | 39346 | 23258 | 26753 | 4587 | 88.5% | -89.0% | -88.3% |
| 5 | Kananga | 214980 | 335090 | 49890 | 87250 | 129885 | 19613 | 59.4% | -61.2% | -60.7% |
| 6 | Bena-Boya | 25456 | 39865 | 7932 | 1259 | 1823 | 362 | 95.1% | -95.4% | -95.4% |
| 7 | Kabinda | 41072 | 66790 | 8884 | 5059 | 7013 | 1130 | 87.7% | -89.5% | -87.3% |
| 8 | Kolwezi | 17295 | 10548 | 1343 | 2040 | 1397 | 203 | 88.2% | -86.8% | -84.9% |
| 9 | Lisala | 20957 | 32764 | 4479 | 2169 | 2924 | 481 | 89.6% | -91.1% | -89.3% |
| 10 | Kenge | 8736 | 20652 | 1889 | 1653 | 2603 | 326 | 81.1% | -87.4% | -82.7% |
| 11 | Idiofa | 10868 | 16667 | 2387 | 1161 | 1622 | 242 | 89.3% | -90.3% | -89.9% |
| 12 | Bukavu | 94523 | 58131 | 7340 | 95250 | 57212 | 7101 | -0.8% | -1.6% | -3.2% |
| 13 | Mweka | 28392 | 52095 | 6871 | 2994 | 4690 | 745 | 89.5% | -91.0% | -89.2% |
| 14 | Mwene-Ditu | 27694 | 52532 | 7328 | 14860 | 26315 | 3601 | 46.3% | -49.9% | -50.9% |
| 15 | Ilebo | 28991 | 43713 | 5859 | 2872 | 3787 | 537 | 90.1% | -91.3% | -90.8% |
| 16 | Bulungu | 7839 | 12953 | 1322 | 1327 | 1752 | 201 | 83.1% | -86.5% | -84.8% |
| 17 | Likasi | 67372 | 28312 | 6147 | 33069 | 13608 | 2979 | 50.9% | -51.9% | -51.5% |
| 18 | Kalemi | 109496 | 44203 | 7252 | 13046 | 5622 | 913 | 88.1% | -87.3% | -87.4% |
| 19 | Kasongo-Lunda | 21240 | 33023 | 7171 | 1659 | 2187 | 419 | 92.2% | -93.4% | -94.2% |
| 20 | Kamonia | 10866 | 21913 | 2783 | 1086 | 1330 | 242 | 90.0% | -93.9% | -91.3% |
| 21 | Demba | 18009 | 36414 | 3948 | 2753 | 4702 | 703 | 84.7% | -87.1% | -82.2% |
| 22 | Butembo | 10863 | 6185 | 828 | 10758 | 6201 | 836 | 1.0% | 0.2% | 1.0% |
| 23 | Gandajika | 21977 | 14649 | 1962 | 2405 | 1367 | 193 | 89.1% | -90.7% | -90.1% |
| 24 | Muanda | 5283 | 8542 | 1202 | 479 | 644 | 95 | 90.9% | -92.5% | -92.1% |
| 25 | Uvira | 34566 | 35518 | 4885 | 2109 | 2319 | 347 | 93.9% | -93.5% | -92.9% |
| 26 | Mbanza-Ngungu | 9895 | 7897 | 795 | 762 | 508 | 58 | 92.3% | -93.6% | -92.7% |
|  | **Total** | **2322610** | **2807419** | **435296** | **1379607** | **1547582** | **252265** | **40.6%** | **-44.9%** | **-42.0%** |

**Supplementary Table 7 |** Comparison of the exposed population for Bukavu (using the hazard zones of 2020; cf. **Methods, ‘Estimating the exposed population’**) based on JRC GHS population estimates for 2018^8^ data, WorldPop population estimates for 2018^66,67^ and population estimates directly derived from detailed Census data for 2018^70^. Relative errors are calculated as compared to the JRC GHS results.

| Exposed population | | | |
| --- | --- | --- | --- |
|  | Within 100 m of UG | Potential Expansion zone | Expected expansion zone |
| JRC GHS 2018 | 91419 | 56157 | 7078 |
| Worldpop 2018 | 92345 | 55595 | 6881 |
| Census data 2018 | 112329 | 75284 | 9861 |
| Relative errors | | | |
|  | Within 100 m of UG | Potential Expansion zone | Expected expansion zone |
| Worldpop 2018 | 1.0% | -1.0% | -2.8% |
| Census data 2018 | 22.9% | 34.1% | 39.3% |
